# Impact of preoperative lumbar paraspinal muscle quality on the prognosis of open pedicle screw fixation for thoracolumbar fractures

**DOI:** 10.3389/fsurg.2026.1759202

**Published:** 2026-06-04

**Authors:** Hao Liu, Yan Gong, Yang Shen, Moshan Wen, Zhen Kuang, Mai Wang, Yufeng Huang, Jintao Liu, Zhensong Yao, Jianchao Cui

**Affiliations:** 1 Department of Cervical Spondylosis and Spinal Orthopedics, The First Affiliated Hospital of Guangzhou University of Chinese Medicine, Guangzhou, China; 2 Department of Orthopedics and Traumatology, Suzhou TCM Hospital Affiliated to Nanjing University of Chinese Medicine, Suzhou, China; 3 School of Integrated Traditional Chinese and Western Medicine, Nanchang Medical College, Nanchang, China

**Keywords:** fat infiltration, open pedicle screw fixation, paraspinal muscles, prognosis, thoracolumbar fractures

## Abstract

**Objective:**

To investigate the association of preoperative paraspinal muscle quality (quantified by fat infiltration) on the clinical and radiographic outcomes following open pedicle screw fixation (OPSF) for thoracolumbar fractures.

**Methods:**

This retrospective study analyzed the clinical data of 48 patients with single-segment thoracolumbar fractures who underwent OPSF surgery between January 2021 and December 2023. Patients were stratified into a low-fat group (LFG, FI < 25%, *n* = 26) and a high-fat group (HFG, FI ≥ 25%, *n* = 22) based on the preoperative fat infiltration rate (FI) of paraspinal muscles at the L4/5 level measured on MRI. General clinical data, perioperative indicators, radiographic parameters (anterior vertebral body height ratio - AVBHr, vertebral body angle - VBA, regional kyphosis angle - RKA), and clinical efficacy scores (Visual Analogue Scale - VAS, Oswestry Disability Index - ODI) were compared between groups preoperatively, at 1 month, and 1 year postoperatively.

**Results:**

The groups were comparable in all baseline and perioperative characteristics (*P* > 0.05). The LFG demonstrated significantly better paraspinal muscle parameters at multiple spinal levels (*P* < 0.05). Although the immediate postoperative radiographic correction achieved was similar between groups (*P* > 0.05), the HFG exhibited significantly greater loss of correction in both VBA and RKA at the 1-year follow-up (*P* < 0.05). This difference in correction loss was particularly pronounced in the subgroup of patients with more severe, unstable fractures (AO type A3/A4). No significant differences were found in VAS and ODI scores at any postoperative time point (*P* > 0.05). Complication rates were similar between groups (*P* > 0.05).

**Conclusion:**

Preoperative lumbar paraspinal muscle quality is not associated with the initial surgical reduction but is significantly associated with the long-term maintenance of radiographic correction after OPSF, especially in unstable fracture patterns. Assessment of paraspinal muscle quality could serve as a valuable prognostic tool for surgical planning and patient counseling.

## Introduction

Thoracolumbar fractures (T10–L2) are the most common type of spinal injury, accounting for more than half of all spinal fractures. Their incidence continues to rise with industrial and transportation development (1). These injuries severely impact patients’ motor function and quality of life, imposing a substantial socioeconomic burden due to prolonged treatment needs (2).

While both conservative and surgical management are employed, surgical intervention, particularly open pedicle screw fixation (OPSF), is often preferred for unstable fractures or those with neurological compromise, aiming to restore spinal stability and facilitate early rehabilitation (3, 4). Despite technical advances, complications such as chronic pain, implant failure, and correction loss persist, underscoring the need to identify modifiable factors influencing long-term outcomes (5, 6).

In recent years, the role of paraspinal muscles in spinal disorders has gradually gained attention in academic circles (7, 8). As the core muscle group is responsible for maintaining the dynamic stability of the spine, the morphology and functional status of the paraspinal muscles directly affect the load distribution of the spine and the postoperative biomechanical environment (9), and its quality is closely related to the occurrence, development, and treatment outcomes of patients with spinal diseases. To quantitatively assess the role of paraspinal muscles, researchers have used imaging techniques such as CT and MRI to measure parameters such as the cross-sectional area and fat infiltration rate of paraspinal muscles (10). Additionally, degenerative features such as paraspinal muscle atrophy and fat infiltration are significantly associated with the occurrence of spinal degenerative diseases and the risk of postoperative complications (11, 12). The latest research further revealed that in patients with thoracolumbar fractures who undergo open or percutaneous pedicle screw internal fixation, open surgery may affect treatment efficacy because of an increase in the rate of paravertebral muscle fat infiltration after surgery (13).

However, the specific impact of preoperative paraspinal muscle quality on outcomes after OPSF for thoracolumbar fractures remains underexplored. This retrospective study aimed to clarify this relationship by analyzing the association between preoperative lumbar paraspinal muscle FI and postoperative clinical and radiographic results.

## Materials and methods

### Participants

This study included clinical data from 48 patients with thoracolumbar single-segment fractures (T11-L2). Patients were divided into a low-fat group (LFG, *n* = 26) and a high-fat group (HFG, *n* = 22) based on the preoperative fat infiltration rate (FI) of the paraspinal muscles at the L4/5 disc level on MRI, using a cut-off value of 25% (LFG: FI < 25%; HFG: FI ≥ 25%). This threshold was adopted from a previous study by Li et al. (14). Inclusion Criteria: ①Age between 18 and 65 years; ②Acute, single-segment traumatic thoracolumbar fracture; ③Underwent OPSF based on the following surgical indications (see below); ④Availability of complete preoperative MRI and follow-up data (clinical and radiographic) at baseline, 1 month, and 1 year postoperatively. Surgical Indications (All patients met at least one): ①Neurological deficit; ②Significant spinal canal compromise (> 30%); ③Severe vertebral body height loss (> 50%); ④Local kyphosis angle > 25°; ⑤Unstable fracture pattern (AO type B or C); ⑥Failure of conservative management with persistent severe pain. Exclusion Criteria: ①History of previous spinal surgery; ②Pathological fractures (osteoporosis, tumor, infection); ③Polytrauma; ④Requirement for additional spinal decompression or corrective osteotomy; ⑤Multi-level thoracolumbar fractures. Incomplete clinical or imaging data. This study protocol was approved by the Institutional Review Board of the First Affiliated Hospital of Guangzhou University of Traditional Chinese Medicine. All participants signed informed consent forms prior to the study and agreed to have their imaging data used for publication.

### Surgical procedure

After successful anesthesia, the patient was placed in the prone position, with the trunk elevated via arch-shaped pads to maintain the thoracolumbar region in a state of mild hyperextension. Reduction was achieved using a combination of three techniques: (1) positioning reduction via prone hyperextension; (2) manual postural reduction by applying gentle pressure over the kyphotic apex; and (3) ligamentotaxis achieved by rod distraction after screw placement. Preoperatively, the fractured vertebra and adjacent vertebrae were identified through localization, their body surface projections were marked, and manual reduction was performed at the thoracolumbar fracture site. Routine disinfection and sterile draping were conducted, followed by a longitudinal incision along the posterior midline. The skin, subcutaneous tissue, and deep fascia were incised layer by layer; after adequate hemostasis, the bilateral laminae and facet joints of the fractured vertebra and adjacent vertebrae were exposed. All patients underwent short-segment pedicle screw fixation spanning one level above and one level below the fractured vertebra (i.e., a total of four pedicle screws: two screws in the vertebra above and two screws in the vertebra below). Screws were not placed into the fractured vertebra itself, and intermediate screws (i.e., screws at the fracture level) were not used. The pedicle screw entry point was determined as the intersection of the upper edge of the transverse process and the lateral side of the facet joint. An awl, hard probe, and soft probe were used sequentially for preparation, after which guide pins were inserted. C-arm fluoroscopy was performed to confirm the satisfactory position of the guide pins, and appropriately sized pedicle screws were inserted one by one. Connecting rods of matching length were selected, prebent according to the physiological curvature of the spine, and placed; the nuts were installed and tightened in sequence. Distraction force was applied through the rods to further restore vertebral body height via ligamentotaxis. Refluoroscopy was conducted to confirm the good position of the internal fixators and satisfactory vertebral reduction. The surgical wound was subsequently irrigated with a large amount of normal saline, a negative-pressure drainage tube was placed, and the incision was sutured layer by layer with a tight approximation.

### Perioperative management

A single dose of antibiotics was administered via intravenous infusion on the day before surgery and on the day of surgery. In the morning and afternoon following surgery, an additional single dose of antibiotics was administered. If the surgery lasted longer than 3 h, an additional single dose of antibiotics was administered during the procedure. On the first postoperative day, the patient was guided to perform bedside self-turning and lower limb functional exercises, and lumbar and thoracic muscle functional exercises were gradually initiated under the protection of a thoracolumbosacral orthosis (TLSO). Once the drainage volume was less than 50 mL after 24 h, the drainage tube was removed. After the x-ray film was reviewed, the patient was instructed to wear the brace and gradually begin walking off the bed. Patients were required to wear the TLSO brace at all times except when lying supine for the first 3 months postoperatively. Thereafter, gradual weaning from the brace was permitted over the following 4–6 weeks based on radiographic healing and clinical assessment.

### Radiographic analyses and data collection

**Figure 1:**
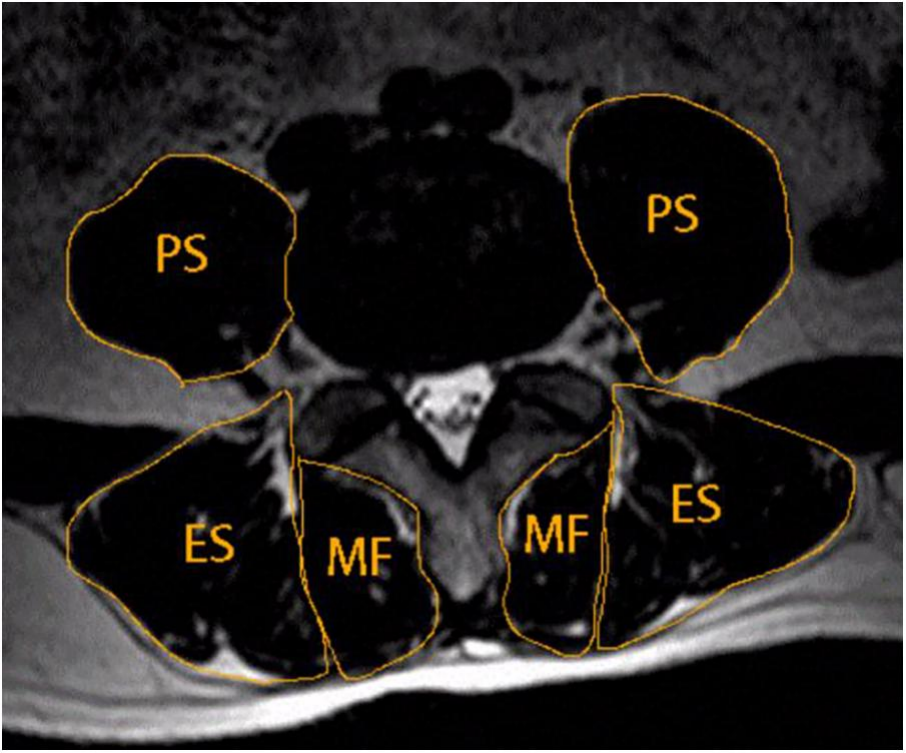
Measurement of the cross-sectional area of paraspinal muscles.

Data were collected for two groups of patients, including sex, age, body mass index (BMI), fracture segment, AO fracture classification, surgical duration, intraoperative blood loss, postoperative complications, and length of hospital stay. Midline images of each intervertebral disc level from the T2-weighted transverse sections of preoperative MR scans were extracted for the L3–S1 segments, and measurements were taken of the bilateral paraspinal muscles (including the PS, ES, and MF). The L4/5 disc level was selected as the primary measurement site for fat infiltration (FI) based on the study by Han et al. (15), which used the same level (L4) to measure multifidus FI with a 25% cutoff value. The extracted images were imported into ImageJ software, and the cross-sectional area (CSA) of the muscles was measured by manually outlining the fascia boundaries (Figure 1). Thresholding techniques were used to distinguish fat tissue within the regions of interest (ROIs) of the paraspinal muscles, and the fat area within each paraspinal muscle ROI was defined as fat CSA. Relative cross-sectional area (rCSA) = muscle CSA/L3 vertebral body CSA, functional cross-sectional area (fCAS) = muscle CSA - fat CSA, relative functional cross-sectional area (rfCSA) = muscle fCSA/L3 vertebral body CSA, fat infiltration rate (FI) = fat CSA/muscle CSA, flexor-extensor cross-sectional area ratio (flexor-extensor CSA ratio) = PS CSA/(ES CSA + MF CSA).

**Figure 2:**
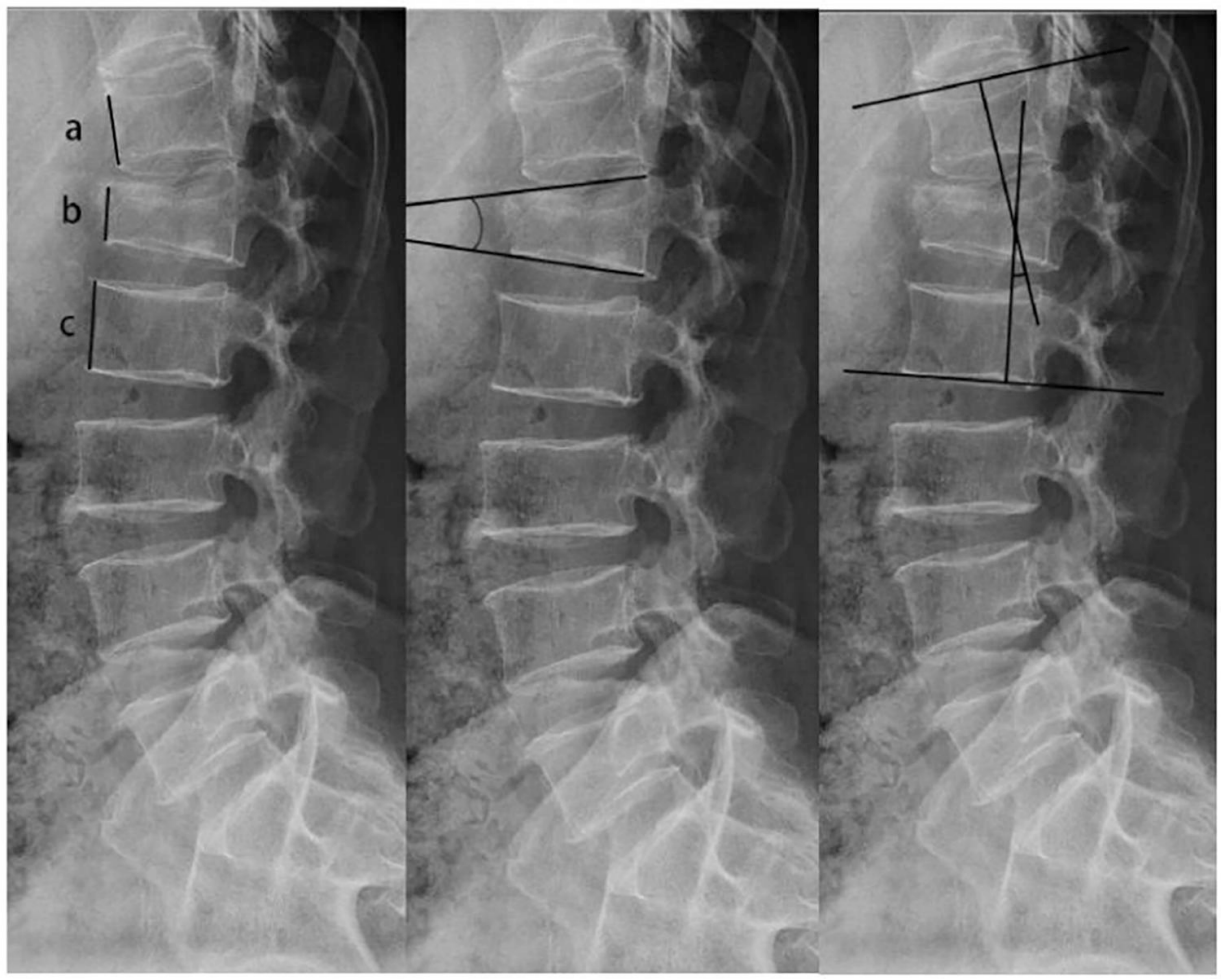
Measurement of anterior vertebral body height (left), vertebral body angle (middle), and regional kyphosis angle (right).

The ratios of the anterior vertebral body height (AVBH), vertebral body angle (VBA), and regional kyphosis angle (RKA) of the injured vertebra were measured at baseline, 1 month post-operatively, and 1 year post-operatively along the *X*-axis (Figure 2). AVBH is the ratio of the anterior vertebral body height to the reference anterior vertebral body height (the reference anterior vertebral body height is the average of the anterior heights of the adjacent upper and lower vertebral bodies), and a smaller ratio indicates a greater difference between the anterior vertebral body height and the ideal height (16). VBA refers to the angle formed by the extended lines of the upper and lower endplates of the injured vertebra; a larger value indicates a greater angle of the injured vertebra and more severe height loss (17). RKA is the angle formed by the perpendicular line between the upper endplate line of the vertebra above the injured vertebra and the lower endplate line of the vertebra below the injured vertebra. A larger value indicates more severe kyphosis of the spine (18). The correction or loss degree (°) of VBA and RKA, i.e., the difference in corresponding angles before surgery, after surgery, and at follow-up, is calculated.

### Statistical analysis

Analyses were performed using SPSS 27.0. Continuous variables are presented as mean ± standard deviation or median (interquartile range). Categorical variables are presented as number (%). Intergroup comparisons were performed using Student's t-test, Mann–Whitney U test, or Chi-square test as appropriate. To assess the potential influence of fracture severity, a subgroup analysis based on AO classification (A1/A2 vs. A3/A4) was performed for key radiographic outcomes. A *P*-value < 0.05 was considered statistically significant.

## Results

### Patient demographics

This study included 48 patients with thoracolumbar single-segment fractures who underwent OPSF. The patients were divided into a low-fat group (*n* = 26) and a high-fat group (*n* = 22) on the basis of fat infiltration rate. There were no statistically significant differences between the two groups in terms of sex, age, BMI, fracture segment, or fracture classification (*P* > 0.05, Table 1).

**Table 1:** Demographic and clinical data of the different groups.

Parameters	LFG	HFG	*P* value
	(*n* = 26)	(*n* = 22)	
Age (year)	45.6 ± 13.6	53.6 ± 6.8	0.077
Gender (Male/Female)	16/10	8/14	0.082
BMI (kg/m²)	24.1 ± 3.3	24.1 ± 3.1	0.906
fractured segment			0.7534
T11	0	1	
T12	4	8	
L1	13	10	
L2	9	3	
AO classification			0.178
A1/A2	14 (53.8%)	7 (31.8%)	
A3/A4	12 (46.2%)	15 (68.2%)	

### Analysis of perioperative data

No cases of implant fracture or vertebral cutting were observed in either group during internal fixation surgery. There were no significant differences between the two groups in terms of surgical duration, intraoperative bleeding, or length of hospital stay (*P* > 0.05, Table 2).

**Table 2:** Comparison of perioperative data between the two groups.

Parameters	LFG	HFG	*P* value
	(*n* = 26)	(*n* = 22)	
Operation time (min)	162.2 ± 56.2	170.0 ± 62.9	0.739
Blood loss (mL)	110.8 ± 51.8	130.0 ± 103.8	0.922
Length of hospital stay (d)	12.4 ± 5.4	12.2 ± 5.4	0.089

### Analysis of paraspinal muscle parameters

At the L3/4 intervertebral disc level, the ES rfCSA, MF rCSA, MF rfCSA, and PS CSA values in the low-fat group were significantly greater than those in the high-fat group (*P* < 0.05). There was no statistically significant difference between the two groups in terms of the ES rCSA and flexor-extensor CSA ratio (*P* > 0.05). At the L4/5 intervertebral disc level, the ES rfCSA, MF rfCSA, PS CSA, and flexor-extensor CSA ratio were significantly greater in the low-fat group than in the high-fat group (*P* < 0.05), whereas there were no statistically significant differences between the two groups in terms of the ES rCSA and MF rCSA (*P* > 0.05). At the L5/S1 intervertebral disc level, the low-fat group had significantly greater MF rfCSA, PS CSA, and flexor-extensor CSA ratio than did the high-fat group (*P* < 0.05), whereas there were no statistically significant differences between the two groups in terms of the ES rCSA, ES rfCSA, and MF rCSA (*P* > 0.05, Table 3).

**Table 3:** Comparison of paraspinal muscle parameters between the two groups.

Parameters	LFG	HFG	*P* value
	(*n* = 26)	(*n* = 22)	
L3/4			
ESrCSA	2.63 ± 2.60	1.88 ± 0.49	0.069
ESrfCSA	2.43 ± 2.61	1.48 ± 0.48	0.001
MFrCSA	0.78 ± 0.17	0.65 ± 0.18	0.010
MFrfCSA	0.65 ± 0.17	0.44 ± 0.15	< 0.001
PSCAS	2158 ± 563.6	1503 ± 604.9	< 0.001
Flexor-extensor CSA ratio	0.43 ± 0.11	0.37 ± 0.14	0.095
L4/5			
ESrCSA	1.57 ± 0.36	1.60 ± 0.40	0.833
ESrfCSA	1.36 ± 0.34	1.12 ± 0.42	0.034
MFrCSA	1.04 ± 0.18	0.92 ± 0.21	0.052
MFrfCSA	0.85 ± 0.15	0.60 ± 0.18	< 0.001
PSCAS	2668 ± 645.5	2407 ± 2170	0.005
Flexor-extensor CSA ratio	0.62 ± 0.13	0.60 ± 0.55	0.002
L5/S1			
ESrCSA	0.95 ± 0.31	1.12 ± 0.47	0.142
ESrfCSA	0.74 ± 0.30	0.65 ± 0.34	0.328
MFrCSA	1.10 ± 0.30	1.02 ± 0.28	0.350
MFrfCSA	0.86 ± 0.32	0.59 ± 0.22	0.001
PSCAS	2560 ± 724.8	1935 ± 499.0	0.001
Flexor-extensor CSA ratio	0.76 ± 0.21	0.59 ± 0.14	0.001

### Analysis of lumbar imaging parameters

**Figure 3:**
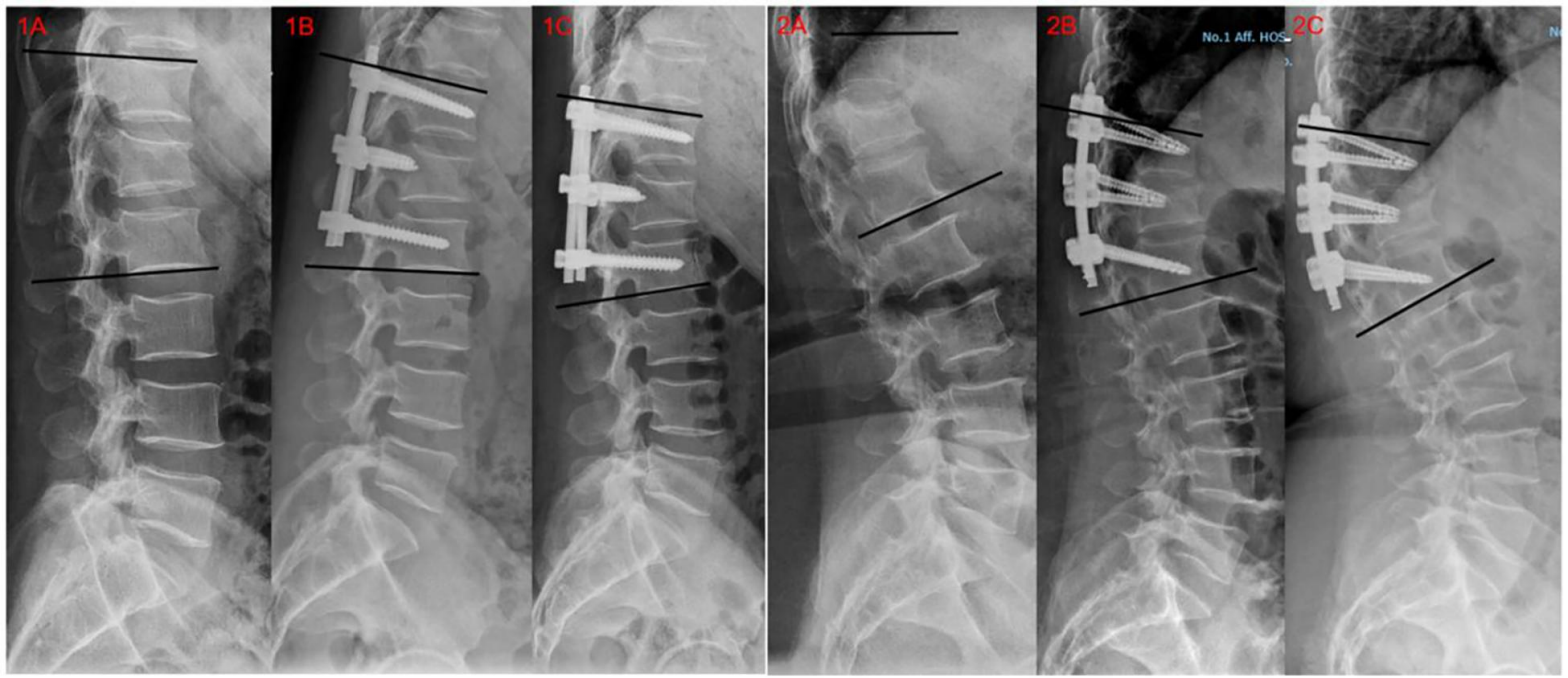
Example of a patient in the low-fat group: **(1A)**. Preoperative x-ray revealed an RKA of 16.9°; **(1B)**. One-month post-operative follow-up x-ray revealed RKA of 13.9°; **(1C)**. One-year post-operative follow-up x-ray revealed an RKA of 13.4°, with a loss of RKA correction of 0.5°. Example of a patient in the high-fat group: **(2A)**. Preoperative x-ray revealed an RKA of 28.3°; **(2B)**. One-month post-operative follow-up x-ray revealed RKA of 23 °C; **(2C)**. One-year post-operative follow-up x-ray revealed RKA of 32.7°, with a loss of RKA correction of 9.7°.

Preoperative radiographic parameters (AVBHr, VBA, and RKA) were comparable between the two groups (*P* > 0.05). The degree of correction achieved at 1 month postoperatively was also similar between the LFG and HFG (*P* > 0.05). However, at the 1-year follow-up, the HFG exhibited significantly greater loss of correction in both VBA (2.21 ± 1.43° vs. 1.16 ± 1.29°, *P* = 0.011) and RKA (4.20 ± 4.21° vs. 1.74 ± 2.19°, *P* = 0.019) compared to the LFG (Table 4). Subgroup analysis of patients with more severe, unstable fractures (AO type A3/A4) revealed that the difference in correction loss between the HFG and LFG was even more pronounced (VBA loss: *P* = 0.005; RKA loss: *P* = 0.008). These findings indicate that while initial fracture severity and surgical reduction were similar, patients with poorer preoperative paraspinal muscle quality (higher fat infiltration) experienced significantly greater long-term loss of radiographic correction, particularly in cases of severe fracture morphology. Two classic cases of thoracolumbar fractures are presented below (Figure 3).

**Table 4:** Comparison of imaging data between the two groups.

Parameters	LFG	HFG	*P* value
	(*n* = 26)	(*n* = 22)	
Preoperative			
AVBHr	0.72 ± 0.13	0.70 ± 0.15	0.672
VBA (°)	14.81 ± 5.54	14.61 ± 5.73	0.900
RKA (°)	12.35 ± 6.72	15.36 ± 8.60	0.181
1-month Postoperative			
VBA Correction (°)	6.58 ± 5.38	5.41 ± 5.72	0.927
RKA Correction (°)	5.12 ± 5.99	7.98 ± 7.11	0.363
1-year Postoperative			
VBA Correction Loss (°)	1.16 ± 1.29	2.21 ± 1.43	0.011
RKA Correction Loss (°)	1.74 ± 2.19	4.20 ± 4.21	0.019
Subgroup analysis (AO A3/A4 only)	(*n* = 12)	(*n* = 15)	
VBA Correction Loss (°)	1.05 ± 1.10	2.65 ± 1.50	0.005
RKA Correction Loss (°)	1.50 ± 1.78	5.60 ± 4.50	0.008

### Analysis of clinical efficacy between the two groups

There was no statistically significant difference between the two groups in terms of the VAS and ODI scores before surgery, one month after surgery, or one year after surgery (*P* > 0.05, Table 5).

**Table 5:** Comparison of clinical efficacy data between the two groups.

Parameters	LFG	HFG	*P* value
	(*n* = 26)	(*n* = 22)	
Preoperative VAS	6.8 ± 1.0	6.9 ± 0.8	0.852
Preoperative ODI	67.5 ± 6.2	68.0 ± 6.7	0.775
1-mo post-op-VAS	3.2 ± 0.9	2.9 ± 0.7	0.283
1-mo post-op-ODI	40.1 ± 4.4	38.0 ± 4.1	0.060
1-yr postop-VAS	0.4 ± 0.6	0.4 ± 0.5	0.999
1-yr postop-ODI	11.8 ± 2.1	11.9 ± 2.2	0.798

### Complications

In terms of postoperative complications, one case of screw fracture occurred in the low-fat group. However, overall, there was no significant difference between the two groups in terms of the incidence of postoperative complications (*P* > 0.05). Although there was an isolated case of screw fracture in the low-fat group, it did not have a significant impact on the overall comparison of postoperative complications between the two groups.

## Discussion

### Interpretation of principal findings

This study demonstrates that preoperative lumbar paraspinal muscle quality, quantified by fat infiltration (FI), is a significant independent factor influencing the long-term maintenance of radiographic correction after open pedicle screw fixation (OPSF) for thoracolumbar fractures. The core findings reveal a critical dissociation: patients with higher FI (the high-fat group, HFG) experienced significantly greater loss of sagittal correction (VBA and RKA) at the 1-year follow-up, despite having achieved a degree of immediate postoperative correction comparable to that of the low-fat group (LFG). Notably, this detrimental effect of poor muscle quality was markedly amplified in the subgroup of patients with more severe, unstable fracture patterns (AO type A3/A4). These results underscore that while surgical technique determines initial reduction, the integrity of the paraspinal muscles is pivotal for sustaining that correction over time, providing a novel biomechanical perspective on post-traumatic deformity progression (19, 20).

### Addressing methodology and confounding factors

It is important to contextualize these findings within our methodological framework. All patients underwent surgery based on explicit, objective indications aligned with established guidelines, ensuring that the observed outcomes are related to patient-specific factors rather than variable surgical criteria (3, 4). Furthermore, we actively addressed the potential confounding role of fracture severity. Although the HFG contained a higher proportion of severe (A3/A4) fractures—a baseline imbalance acknowledged in our analysis—the subsequent subgroup analysis confirmed that the negative impact of high FI was most pronounced within this very subgroup. This reinforces the conclusion that muscle quality is a potent prognostic modifier, particularly in mechanically demanding, unstable injuries.

### Biomechanical explanations and the “load-sharing” theory

The observed divergence between immediate correction and long-term maintenance is central to a biomechanical interpretation. The paraspinal muscles, especially the multifidus (MF), are fundamental to the spine's dynamic stability (21). Our data show the LFG possessed significantly greater relative functional cross-sectional area (rfCSA) of the MF across multiple levels, indicating superior muscle contractile reserve. The MF's unique architecture, rich in fatigue-resistant type I fibers, allows it to function as a “dynamic ligament,” providing continuous segmental stabilization against flexion and rotational forces (22). High FI represents a degeneration of this contractile tissue, impairing muscle strength and endurance. Consequently, a spine reliant on degenerated musculature cannot effectively offload stress from the posterior pedicle screw construct. This leads to abnormal stress concentration at the bone-implant interface, accelerating screw loosening and subsequent correction loss—a pathophysiology perfectly aligned with the “load-sharing” theory of spinal instrumentation (23).

The finding that paraspinal muscle differences were most significant at the L4/5 level further supports this biomechanical rationale. As a major transitional zone subject to high shear and rotational forces, the L4/5 segment places exceptional demands on muscular stabilization (12). Degeneration here may therefore create a critical “weak link,” predisposing to earlier and more substantial mechanical failure and correction loss (24).

### Interaction between paraspinal muscle degeneration and bone mineral density

It is important to acknowledge the established relationship between paraspinal muscle fat infiltration and osteoporosis. Previous studies have demonstrated that muscle degeneration and low bone mineral density (BMD) often co-exist, particularly in older adults and postmenopausal women, and both contribute independently to poor outcomes after spinal instrumentation. Degenerative muscle may reduce mechanical loading on the vertebral bodies, leading to disuse osteoporosis, while osteoporotic bone provides poorer screw purchase, further increasing the risk of correction loss. Unfortunately, we were unable to assess BMD in our cohort due to the lack of preoperative CT or DXA data. Therefore, we cannot exclude the possibility that undetected differences in bone quality between the low-fat and high-fat groups may have partly contributed to the observed differences in correction loss. Future studies should incorporate BMD evaluation (e.g., CT Hounsfield units) to determine whether paraspinal muscle quality has an independent effect on radiographic outcomes beyond bone density.

### The discrepancy between radiographic and clinical outcomes

A key observation is the lack of significant difference in short-term clinical scores (VAS, ODI) between groups, despite clear radiographic deterioration in the HFG. This discrepancy may be attributed to several factors. First, the 1-year follow-up may be insufficient for the biomechanical compromise to manifest as debilitating pain or functional decline, a process which may follow a delayed course. Second, the body may employ compensatory mechanisms from adjacent spinal segments or altered kinematics to temporarily mask symptoms. This highlights a crucial clinical insight: relying solely on short-term patient-reported outcomes may overlook significant underlying structural deterioration. Long-term radiographic surveillance remains essential, especially in patients with identifiable risk factors such as poor preoperative muscle quality (25). The clinical relevance of radiographic correction loss should not be underestimated. Progressive kyphosis, even if initially asymptomatic, may lead to sagittal imbalance, chronic mechanical pain, adjacent segment degeneration, and even implant-related complications (e.g., screw loosening or rod fracture) over the long term. Although no significant differences in VAS or ODI were observed at 1 year, the delayed consequences of correction loss may become clinically manifest beyond this timeframe as compensatory mechanisms gradually fail. Therefore, for patients with poor preoperative paraspinal muscle quality, we recommend a more vigilant postoperative surveillance strategy (e.g., regular radiographic follow-up at 2 and 5 years) and consideration of adjunctive measures such as preoperative core muscle strengthening, extended bracing, or, in selected cases with severe muscle degeneration, more rigid fixation constructs (e.g., longer segment fixation or cement-augmented screws).

### Limitations and future directions

This study has limitations that must be acknowledged. Its retrospective design and modest sample size (*n* = 48) limit causal inferences, increase the risk of type II error, and may leave residual confounding. Therefore, our findings should be interpreted as exploratory and hypothesis-generating rather than confirmatory. The imbalance in fracture severity between groups (more A3/A4 in high-fat group) and the lack of multivariate adjustment due to small sample size are additional limitations. Potential selection bias may also exist due to the single-center, non-randomized design. We assessed only a preoperative snapshot of muscle status; serial postoperative MRI would be necessary to dynamically link muscle changes to correction loss. Furthermore, we did not compare surgical approaches (e.g., minimally invasive versus open), which may differentially affect paraspinal muscle integrity. Other limitations include the absence of bone quality assessment (e.g., CT Hounsfield units) and the relatively short 1-year follow-up, which may not capture delayed clinical consequences of correction loss. Future prospective studies should: (1) establish causality through longitudinal muscle imaging, (2) investigate whether preoperative rehabilitation to improve muscle quality can enhance long-term outcomes, and (3) compare how different surgical techniques modulate the risk associated with poor muscle quality.

## Conclusion

In conclusion, preoperative fat infiltration of the lumbar paraspinal muscles is a key prognostic factor for the loss of sagittal correction after OPSF for thoracolumbar fractures. It does not impede the initial surgical reduction but critically compromises the spine's capacity to maintain it, particularly in unstable fracture patterns. We recommend integrating the assessment of paraspinal muscle quality into the preoperative evaluation. Identifying at-risk patients could improve prognostic stratification, guide patient counseling, and stimulate the development of targeted pre- and postoperative rehabilitation strategies aimed at preserving spinal stability and improving long-term surgical outcomes.

## Data Availability

The original contributions presented in the study are included in the article/Supplementary Material, further inquiries can be directed to the corresponding authors.
